# Epigenetic Control of the *foxp3* Locus in Regulatory T Cells 

**DOI:** 10.1371/journal.pbio.0050038

**Published:** 2007-01-30

**Authors:** Stefan Floess, Jennifer Freyer, Christiane Siewert, Udo Baron, Sven Olek, Julia Polansky, Kerstin Schlawe, Hyun-Dong Chang, Tobias Bopp, Edgar Schmitt, Stefan Klein-Hessling, Edgar Serfling, Alf Hamann, Jochen Huehn

**Affiliations:** 1 Experimentelle Rheumatologie, Charité Universitaetsmedizin Berlin, Berlin, Germany; 2 Epiontis, Berlin, Germany; 3 Deutsches Rheuma-Forschungszentrum, Berlin, Germany; 4 Institut für Immunologie, Johannes-Gutenberg-Universität, Mainz, Germany; 5 Abteilung für Molekulare Pathologie, Pathologisches Institut, Wuerzburg, Germany; National Jewish Medical and Research Center, United States of America

## Abstract

Compelling evidence suggests that the transcription factor Foxp3 acts as a master switch governing the development and function of CD4^+^ regulatory T cells (Tregs). However, whether transcriptional control of Foxp3 expression itself contributes to the development of a stable Treg lineage has thus far not been investigated. We here identified an evolutionarily conserved region within the *foxp3* locus upstream of exon-1 possessing transcriptional activity. Bisulphite sequencing and chromatin immunoprecipitation revealed complete demethylation of CpG motifs as well as histone modifications within the conserved region in ex vivo isolated Foxp3^+^CD25^+^CD4^+^ Tregs, but not in naïve CD25^−^CD4^+^ T cells. Partial DNA demethylation is already found within developing Foxp3^+^ thymocytes; however, Tregs induced by TGF-β in vitro display only incomplete demethylation despite high Foxp3 expression. In contrast to natural Tregs, these TGF-β–induced Foxp3^+^ Tregs lose both Foxp3 expression and suppressive activity upon restimulation in the absence of TGF-β. Our data suggest that expression of Foxp3 must be stabilized by epigenetic modification to allow the development of a permanent suppressor cell lineage, a finding of significant importance for therapeutic applications involving induction or transfer of Tregs and for the understanding of long-term cell lineage decisions.

## Author Summary

Regulatory T cells play a pivotal role in the maintenance of self-tolerance within the immune system by preventing autoimmunity or excessive activation of the T cells that respond to pathogens (naïve and effector T cells). They differentiate within the thymus, but can also be de novo induced in the rest of the body. Mechanisms determining development of a stable regulatory T cell lineage are unknown. Our study provides evidence for a critical role of epigenetic modifications in the locus coding for the forkhead transcription factor Foxp3, which acts as a master switch controlling regulatory T cell development and function: An evolutionarily conserved region within the non-coding part of the gene contains CpG motifs, which are completely demethylated in regulatory T cells, but methylated in naïve and effector T cells, whereas we observed an inverse occurrence of acetylated histones, another epigenetic chromatin modification. Regulatory T cells induced in vitro—which, in contrast to natural regulatory T cells, do not display a stable regulatory T cell phenotype—display only incomplete DNA demethylation despite high Foxp3 expression. Our data suggest that expression of Foxp3 must be stabilized by epigenetic modification to result in a permanent suppressor cell lineage, a finding of significant importance for therapeutic applications involving induction or transfer of regulatory T cells and for the understanding of long-term cell lineage decisions.

## Abstract 3

The transcription factor Foxp3 is a master switch for the regulatory T cell lineage. The authors show that the Foxp3 locus is epigenetically modified in stable regulatory T cells.

## Introduction

Regulatory T cells (Tregs), which have been shown to play a pivotal role in the maintenance of self-tolerance within the immune system, were described originally as CD4^+^ T cells constitutively expressing CD25 [1]. More recently, the forkhead transcription factor Foxp3 has been shown to be specifically expressed in Tregs and to be a central control element in Treg development and function [2]. Mutation or deletion of the gene encoding Foxp3 causes severe autoimmune disease in mice and humans, due to a failure to generate CD25^+^CD4^+^ Tregs [3,4], whereas ectopic expression of Foxp3 in conventional T cells confers suppressive activity [4,5]. These findings provided compelling evidence that Foxp3 acts as a master switch controlling the development and function of Tregs; however, the molecular mechanisms leading to its induction remain largely unknown. Recently, an initial characterization of the human FOXP3 promoter revealed a basal, T cell–specific promoter containing several NF-AT and AP-1 binding sites, which are positively regulating FOXP3 expression after triggering of the T cell receptor (TCR) [6].

Occurrence of autoimmunity in thymectomized mice provided initial evidence that Foxp3^+^CD25^+^CD4^+^ Tregs are generated as an individual lineage within the thymus [1]. Using mice harboring a GFP-Foxp3 fusion protein-reporter knockin allele, Fontenot et al. could show that Foxp3 expression becomes prominent in CD4 single-positive (SP) thymocytes (∼83% of Foxp3^gfp+^ thymocytes) [7,8]. In addition to the thymic generation of Foxp3^+^ Tregs, peripheral conversion of Foxp3^−^CD25^−^CD4^+^ T cells into Foxp3^+^ Tregs has recently been demonstrated by tolerogenic antigen application in vivo [9–11] or upon activation in the presence of TGF-β in vitro [12–17]. To what extent these induced populations of Tregs acquire a stable phenotype corresponding to that of natural, thymus-derived Tregs is, however, unclear.

An emerging paradigm in understanding the development of stable cellular lineages emphasizes the role of epigenetic mechanisms for the permanent, heritable fixation of distinct gene expression patterns. Molecular mechanisms of epigenetic imprinting include selective demethylation of CpG motifs and histone modifications as shown for cytokine genes [18–20]. Whether Treg differentiation also involves elements of epigenetic regulation has not been studied thus far. We therefore investigated whether epigenetic alterations such as DNA methylation and histone modifications of the *foxp3* locus correlate with Foxp3 expression. The selective association of chromatin remodeling with a stable Treg phenotype suggests a role of epigenetic imprinting in the establishment of a committed regulatory cell type.

## Results

### CD25^+^CD4^+^ Tregs Display a Stable Foxp3 Expression

**Figure 1:**
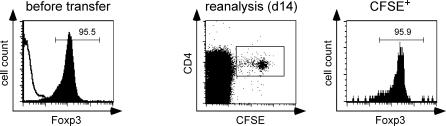
Stable Foxp3 Expression in CD25^+^CD4^+^ Tregs CD25^+^CD4^+^ Tregs were isolated ex vivo from pooled spleen and LN single-cell suspensions, labeled with CFSE, and then transferred into syngenic 8-wk-old recipients (2 × 10^6^/mouse). Before transfer, sorted cells were analyzed for intracellular Foxp3 expression by FACS. Fourteen days after transfer, splenocytes of recipient mice were stained for CD4, CD25, and Foxp3, and analyzed by FACS. About 0.1% of total splenocytes and, accordingly, 3% of total Foxp3^+^ splenocytes were donor derived (CFSE^+^). Representative dot and histogram plots from four independently analyzed mice were selected. Numbers display frequency of cells within indicated populations. Comparable results were obtained for cells isolated from peripheral or mesenteric LNs (unpublished data). The bars in the left and right graphs indicate the marker gate for Foxp3^+^ cells. The box in the middle graph indicates the region that was used to gate for CD4^+^CFSE^+^ cells. These gated cells (CFSE^+^) are depicted in the right graph.

It is assumed that Foxp3^+^CD25^+^CD4^+^ Tregs represent an individual lineage exhibiting a stable phenotype. To prove experimentally the stability of Foxp3 expression in natural Tregs on a cellular level, we adoptively transferred sorted CD25^+^CD4^+^ T cells after CFSE (carboxy fluorescein diacetate succinimide ester) labeling into syngeneic recipients. Fourteen days after transfer, more than 95% of CFSE^+^ cells, including those that had divided once or twice according to loss of CFSE, were still Foxp3^+^ (Figure 1), supporting data from a recent publication using a lymphopenic transfer model [21]. Having shown that Foxp3 is stably expressed in CD25^+^CD4^+^ Tregs, we next asked whether epigenetic modifications of the *foxp3* locus might account for the maintenance of the long-term identity of Foxp3^+^ Tregs.

### Epigenetic Modifications of the *foxp3* Locus in Foxp3^+^CD25^+^CD4^+^ Tregs

**Figure 2:**
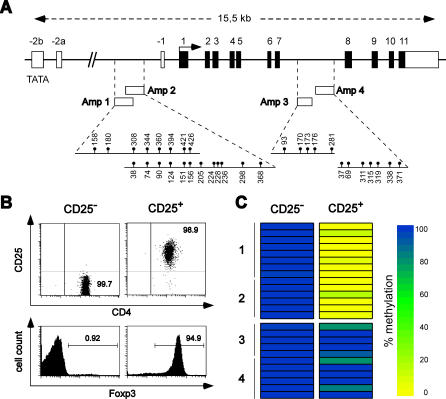
Selectively Demethylated CpG Motifs within the *foxp3* Locus in CD25^+^CD4^+^ Tregs Isolated from Secondary Lymphoid Organs (A) Schematic view on the *foxp3* locus depicts exon-intron structure and position of selected amplicons (Amp 1–4). Shown is the distribution and position of individual CpG motifs within the amplicons. (B) CD25^+^CD4^+^ and CD25^−^CD4^+^ T cells were sorted from spleens and LNs pooled from 20 male BALB/c mice. FACS analysis shows the sort purity (upper panel) and Foxp3 expression in sorted subsets (lower panel). Numbers display frequency of cells within indicated populations. The bars in the lower graphs indicate the marker gate for Foxp3^+^ cells. The vertical and horizontal lines in the upper graphs indicate the quadrant used to identify the CD4^+^CD25^+/−^ subsets. (C) Methylation pattern of selected amplicons of the *foxp3* locus in CD25^+^CD4^+^ Tregs and conventional CD25^−^CD4^+^ T cells. The amplicons are subdivided by horizontal lines each representing an individual CpG motif. ESME software [47] is used for normalization and quantification of methylation signals from sequencing data by calculating ratios of T and C signals at CpG sites. Data are condensed to methylation information at CpG positions forming matrices of consecutive CpGs. The methylation status of individual CpG motifs within the four amplicons is color coded according to the degree of methylation at that site. The color code ranges from yellow (0% methylation) to blue (100% methylation) according to the color scale on the right. CpG motifs from amplicon 2 overlapping with motifs in amplicon 1 were excluded. Due to sequencing problems, the CpG motif 37 from amplicon 4 is not listed. One representative experiment out of two individual experiments is shown.

The lymphoproliferative disorder of scurfy mice is completely rescued by transgenic complementation with a 30.8-kilobase (kb) genomic fragment containing the *foxp3* gene from wild-type mice [22]. This indicates that most key regulatory elements required for proper Foxp3 expression are located within the transgene including the entire gene, as well as 12.5 kb and 2.8 kb of 5′ and 3′ flanking sequences, respectively. We therefore focused our analysis of epigenetic modifications on sequences from the 30.8-kb region and selected specific regions for methylation analysis based on CpG density (Figure 2A): Overlapping amplicons 1 and 2 map upstream from exon-1, amplicons 3 and 4 align to the seventh intron. No CpG-rich regions were observed within the Foxp3 promoter located at the putative 5′ end of exon-2b, 6.1 kb upstream from the first coding exon [6,22].

We sorted CD25^+^CD4^+^ Tregs and conventional CD25^−^CD4^+^ T cells from secondary lymphoid organs of male mice. Male mice were chosen to avoid potential artifacts due to random X chromosome inactivation since Foxp3 is encoded on the X chromosome [22]. As expected, the vast majority of sorted CD25^+^CD4^+^ Tregs were Foxp3^+^, whereas less than 1% of CD25^−^CD4^+^ T cells expressed Foxp3 (Figure 2B).

The methylation status of the *foxp3* locus was analyzed by bisulphite sequencing (see Material and Methods). Interestingly, striking differences between CD25^+^CD4^+^ Tregs and conventional CD25^−^CD4^+^ T cells could be observed. CpG motifs within amplicons 1 and 2 displayed a high degree of methylation (∼100%) within conventional CD25^−^CD4^+^ T cells, but were almost completely demethylated within CD25^+^CD4^+^ Tregs (Figure 2C and Table S1). No significant differences were observed for amplicons 3 and 4, showing that the demethylation process is not a random event, but is confined to defined regions as was recently found for the interleukin 2 (IL-2) promoter [23]. Together, our findings suggest that demethylation of CpG motifs within selected elements of the *foxp3* locus enable stable Foxp3 expression in CD25^+^CD4^+^ Tregs. This view is supported by recent findings with human natural killer (NK) cells, which up-regulated FOXP3 expression in response to IL-2 only after treatment with the demethylating agent 5-aza-2′deoxycytidine, demonstrating that this gene was constitutively repressed in non-Tregs by a mechanism involving DNA methylation [24].

### Differentially Methylated Element of the *foxp3* Locus Is Evolutionarily Conserved and Possesses Transcriptional Activity

The differentially methylated element covered by amplicons 1 and 2 is conserved between mice and humans (77.3% sequence identity). This is not the case for the region covered by amplicons 3 and 4, which essentially showed no signs of differential DNA methylation. In silico analysis of the differentially methylated, conserved region predicts a number of binding sites for transcription factors, including ATF/CREB, C/EBPγ, Elk-1, Ets-1, Evi-1, Foxp3, GATA-4, NFATc, NF-κB, SMAD-4, STAT-1, TCF-4, and TTF1 (Figure S1), indicating that these factors might be involved in the induction of Foxp3 expression in CD25^+^CD4^+^ Tregs.

**Figure 3:**
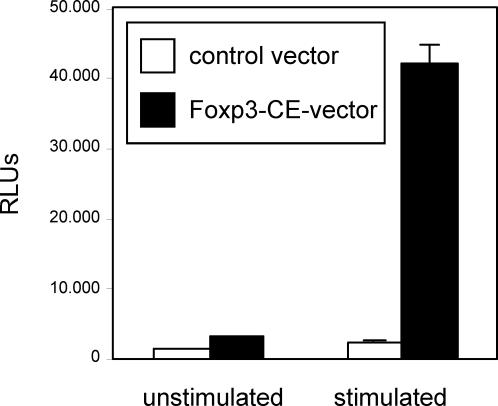
Differentially Methylated, Conserved Element of the *foxp3* Locus Possesses Transcriptional Activity RLM-11–1 cells were transfected with control vector (pGL3 promoter) or Foxp3-CE vector containing the conserved element (CE). Cells were stimulated with PMA for 24 h. Control cells were left unstimulated. Results given are relative luciferase light units (RLUs) normalized for Renilla luciferase activity (mean ± standard deviation; *n* = 3). Results are representative for two independent experiments.

To analyze whether the same region harbors transcriptional activity, we cloned a 1,160–base pair (bp) element containing the differentially methylated element covered by amplicons 1 and 2 into the pGL3 luciferase vector in front of a minimal SV40 promoter. The luciferase vector was transfected into a murine CD4^+^ T cell line, and transfected cells were either left unstimulated or were stimulated with PMA for 24 h, followed by measurement of luciferase activity. PMA treatment mimics part of the signals generated after TCR triggering, which have been shown to be essential for Foxp3 expression [6]. Interestingly, significant luciferase activity was only observed in stimulated cells transfected with the vector containing the conserved element of the *foxp3* locus, but not in cells transfected with the control vector (Figure 3). Similar results showing a 5-fold to 7-fold induction of luciferase activity after stimulation were also obtained with ex vivo isolated CD25^+^CD4^+^ Tregs, albeit much lower transfection efficiencies were achieved with these primary murine T cells (unpublished data). Initial experiments targeting the functional activity of selected transcription factors, for which a binding site in the differentially methylated, conserved element has been predicted, showed a reduced luciferase activity if transcription factors of the STAT family were inhibited by decoy oligonucleotides (unpublished data), confirming recently published data [24]. Together, our data assuredly show that the differentially methylated, conserved element of the *foxp3* locus possesses transcriptional activity.

### Histone Modifications Indicate Opening of the *foxp3* Locus in CD25^+^CD4^+^ Tregs

**Figure 4:**
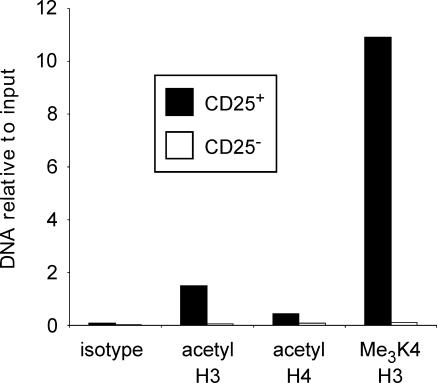
Increased Histone Acetylation and K4 Trimethylation in CD25^+^CD4^+^ Tregs ChIP assays were performed with CD25^+^CD4^+^ Tregs (filled bars) and conventional CD25^−^CD4^+^ T cells (open bars) sorted from spleens and LNs pooled from 20 male BALB/c mice. DNA fragments binding to acetylated or trimethylated histones were immunoprecipitated using antibodies directed against acetylated histone H3, acetylated histone H4, or trimethylated histone H3 at position K4. A rabbit isotype immunoglobulin G (IgG) served as control. Precipitated DNA was quantified by real-time PCR with primers specific for the differentially methylated region of the *foxp3* locus. Sample PCR products were set in relation to input DNA. One representative experiment out of two individual experiments is shown.

DNA demethylation is often linked to acetylation or methylation of histones, other key features of chromatin remodeling [25]. To investigate whether this also holds true for the aforementioned region of the *foxp3* locus, we performed chromatin immunoprecipitation (ChIP) experiments using antibodies specific for acetylated histone H3, acetylated histone H4, and trimethylated lysine 4 of histone H3 (H3K4). Subsequently, the precipitated DNA was used as a template for amplifying the differentially methylated region of the *foxp3* locus by quantitative real-time PCR. Indeed, in CD25^+^CD4^+^ Tregs, the region of interest showed a stronger association with modified histones when compared with conventional CD25^−^CD4^+^ T cells (Figure 4). Major differences were observed for the acetylated and trimethylated histone H3, whereas minor differences were found for the acetylated histone H4. Together, the ChIP data disclose that within conventional CD25^−^CD4^+^ T cells, the *foxp3* locus is packed in a more condensed, inaccessible chromatin structure, whereas it is located within open euchromatin in CD25^+^CD4^+^ Tregs.

### Demethylated CpG Motifs in Developing Foxp3^+^ Thymocytes

**Figure 5:**
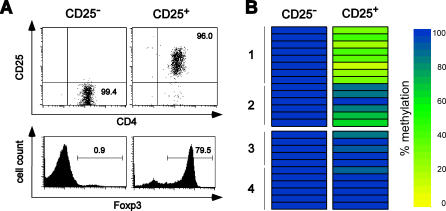
Demethylated CpG Motifs within the *foxp3* Locus in CD25^+^ CD4 SP Thymocytes (A) CD25^+^CD4^+^CD8^−^ and CD25^−^CD4^+^CD8^−^ subsets were sorted from thymocytes pooled from 30 male BALB/c mice. FACS analysis shows purity of sorted subsets (upper panel) and Foxp3 expression in gated CD25^−^ and CD25^+^ subsets of CD4^+^ SP thymocytes (lower panel). Numbers display frequency of cells within indicated populations. The bars in the lower graphs indicate the marker gate for Foxp3^+^ cells. The vertical and horizontal lines in the upper graphs indicate the quadrant used to identify the CD4^+^CD25^+/−^ subsets. (B) Methylation pattern of selected amplicons of the *foxp3* locus in CD25^+^ and CD25^−^ CD4 SP thymocytes. The methylation status of individual CpG motifs within the four amplicons is color coded as described in Figure 2. One representative experiment out of two individual experiments is shown.

Having shown that peripheral CD25^+^CD4^+^ Tregs display a characteristic methylation status of the *foxp3* locus, we next sought to determine whether this also could be observed in developing Tregs. Generation of Foxp3^+^ cells in the thymus occurs preferentially at the CD4 SP stage or during transition to this stage [8]. We therefore isolated CD25^+^ and CD25^−^ subsets of CD4 SP thymocytes from male mice. As expected, approximately 80% of CD25^+^ CD4 SP thymocytes were Foxp3^+^, whereas less than 1% of CD25^−^ CD4 SP thymocytes expressed Foxp3 (Figure 5A). Only CD25^+^, not CD25^−^ CD4 SP thymocytes, displayed demethylated CpG motifs within the regions covered by amplicons 1 and 2, whereas CpG motifs in amplicons 3 and 4 were again fully methylated in both subsets (Figure 5B and Table S1). As expected, in double-negative (DN) and double-positive (DP) thymocytes, which show hardly any Foxp3 expression [7,8], CpG motifs within the regions covered by amplicons 1 and 2 were completely methylated (Table S1), supporting our assumption that Foxp3 expression is developmentally regulated and requires an opening of the *foxp3* locus. When compared to peripheral CD25^+^CD4^+^ Tregs, in which the CpG motifs within amplicons 1 and 2 were almost completely demethylated (mean degree of methylation <3%), CD25^+^ CD4 SP thymocytes showed a clearly reduced degree of demethylated DNA (mean degree of methylation ∼50%) with individual CpG motifs being even completely methylated (Table S1). These stark differences cannot simply be explained by the fact that, among CD25^+^ CD4 SP thymocytes, only 80% are Foxp3^+^ compared to 95% within the peripheral counterpart (Figures 2B and 5A). Rather, it indicates that the locus does not become fully opened until completion of maturation and exit from the thymus.

### Weak CpG Demethylation Correlates with Poor Foxp3 Stability in TGF-β–Induced Tregs

**Figure 6:**
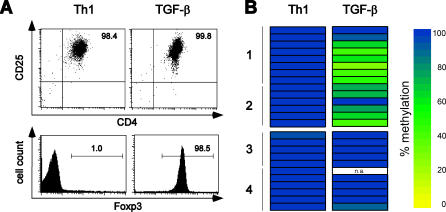
TGF-β–Induced Tregs Harbor Partially Demethylated CpG Motifs within the *foxp3* Locus (A) CD25^−^CD4^+^ T cells sorted from spleens and LNs pooled from 20 male BALB/c mice were cultured for 6 d in the presence of TGF-β. Cells cultured under Th1 conditions were used as control. Within the starting population, less than 1% of the cells were Foxp3^+^ (unpublished data). On day 6, cultured cells were sorted by FACS for CD25^+^ cells. FACS analysis shows the purity of sorted subsets (upper panel) and Foxp3 expression in gated CD25^+^ cells derived from indicated cultures (lower panel). Numbers display frequency of cells within indicated populations. The bars in the lower graphs indicate the marker gate for Foxp3^+^ cells. The vertical and horizontal lines in the upper graphs indicate the quadrant used to identify the CD4^+^CD25^+/−^ subsets. (B) Methylation pattern of selected amplicons of the *foxp3* locus in sorted CD25^+^ cells derived from indicated cultures. The methylation status of individual CpG motifs within the four amplicons is color coded as described in Figure 2. One representative experiment out of two individual experiments is shown. n.a., not analyzed.

**Figure 7:**
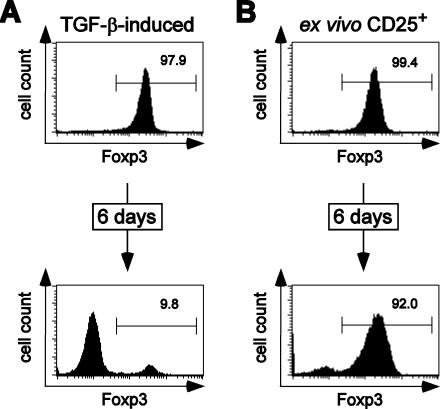
TGF-β–Induced Foxp3^+^ Tregs Loose Foxp3 Expression upon Restimulation (A) CD25^−^CD4^+^ T cells were cultured for 6 d in the presence of TGF-β as described in Figure 6. On day 6, cultured cells were sorted by FACS for CD25^+^ cells and analyzed for intracellular Foxp3 expression by FACS (upper panel). Sorted CD25^+^ cells from the same TGF-β-cultures were restimulated for another 6 d in the absence of TGF-β. On day 6, cultured cells were analyzed for intracellular Foxp3 expression by FACS (lower panel). Numbers display frequency of cells within indicated populations. (B) Ex vivo isolated CD25^+^CD4^+^ Tregs were analyzed for intracellular Foxp3 expression before (upper panel) and after 6 d in vitro culture (lower panel) by FACS. Numbers display frequency of cells within indicated populations. One representative experiment out of two individual experiments is shown.

A critical issue for application of Tregs in therapeutic approaches is the availability of large numbers of cells. Recent publications have reported that conventional CD25^−^CD4^+^ T cells can be converted into Foxp3^+^ Tregs by stimulation in the presence of TGF-β [12–17]. However, the stability and in vivo efficacy of these cells have not been thoroughly tested so far. Analysis of the accessibility of the *foxp3* locus might provide an additional clue, aside from the mere expression of Foxp3, as to the extent to which a permanent conversion into a Treg lineage did occur. We therefore analyzed the methylation status of the *foxp3* locus from CD25^−^CD4^+^ T cells, which had been activated and cultured for 6 d in the presence of TGF-β. On day 6, more than 98% of TGF-β–cultured cells were Foxp3^+^, whereas control cells cultured under Th1 conditions showed only approximately 1% Foxp3 expression (Figure 6A). As expected, within cultured Th1 cells, all analyzed CpG motifs were completely methylated (Figure 6B and Table S1). In contrast, cell culture in the presence of TGF-β led to a clearly visible demethylation of CpG motifs within the region covered by amplicons 1 and 2, whereas CpG motifs in amplicons 3 and 4 again were fully methylated. However, the degree of demethylation was far less pronounced compared to naturally occurring peripheral CD25^+^CD4^+^ Tregs (Figure 2C). This prompted us to investigate whether such a weak degree of CpG demethylation might correlate with persistent expression of Foxp3 in TGF-β–induced Tregs. Therefore, we restimulated TGF-β–cultured Foxp3^+^ cells for another 6 d in the absence of TGF-β followed by the analysis of intracellular Foxp3 expression. As a control, we cultured ex vivo isolated Foxp3^+^CD25^+^CD4^+^ Tregs under comparable conditions. Whereas ex vivo CD25^+^CD4^+^ Tregs maintained high Foxp3 levels after cell culture for 6 d, TGF-β–induced Tregs have lost Foxp3 expression during the 6-d restimulation period to variable degrees, and initial results point toward an almost complete loss of the partial demethylation of the CpG motifs within amplicons 1 and 2 in the restimulated TGF-β cultures (Figure 7, and unpublished data). To rule out selective outgrowth of Foxp3^−^ cells or enhanced cell death of Foxp3^+^ cells during restimulation, we performed TGF-β cultures with CD25^−^CD4^+^ T cells from GFP-Foxp3 reporter mice [7], which allowed sorting of TGF-β–induced Foxp3^+^ Tregs to a purity greater than 99% before restimulation. In other control experiments, we either labeled TGF-β–induced Tregs with CFSE before restimulation or did spiking experiments with Foxp3^−^ T cells. In none of these control experiments was outgrowth of Foxp3^−^ cells or massive cell death observed (unpublished data), confirming our previous assumption that Foxp3 expression in TGF-β–induced Tregs is lost during restimulation in the absence of TGF-β. Importantly, loss of Foxp3 expression was strictly associated with loss of suppressive activity when tested in in vitro proliferation assays (Figure S2). Viewed as a whole, our data strongly suggest that complete demethylation of CpG motifs within the *foxp3* locus is required to stabilize both Foxp3 expression and suppressive capacity.

## Discussion

The forkhead box transcription factor Foxp3 has been identified as a specific molecular marker for Tregs, and its expression is essential for programming Treg development and function [2]. Although it is widely accepted that Foxp3^+^ Tregs represent a stable population mainly generated as a separate lineage in the thymus, conclusive data on the molecular mechanisms maintaining stable Foxp3 expression are not available. We here provide evidence that epigenetic modifications of the *foxp3* locus are required to enable long-term identity of Foxp3^+^ Tregs.

We have identified an element within the 5′ untranslated region of the *foxp3* locus, TSDR (Treg-specific demethylated region), which displays demethylated CpG motifs both in developing thymic as well as in mature, peripheral murine Foxp3^+^CD25^+^CD4^+^ Tregs. Interestingly, the differentially methylated element is evolutionarily conserved. Preliminary analyses using cells from human peripheral blood also showed a differential methylation of CpG motifs within the conserved element of the *foxp3* locus when conventional CD25^−^CD4^+^ T cells and CD25^high^CD4^+^ Tregs were compared, implying that this region and its epigenetic regulation is of functional importance (unpublished data).

In addition to DNA demethylation, acetylated histones H3 and H4 as well as trimethylated histone H3 were associated with the conserved region in CD25^+^CD4^+^ Tregs, but not in conventional CD25^−^CD4^+^ T cells. Similar histone modifications have frequently been reported to concur with DNA demethylation, e.g., as described for the loci encoding the active cytokines interferon-γ (IFN-γ) and IL-4 in differentiated Th1 and Th2 cells, respectively [19,20]. These data suggest that in terminally differentiated Tregs, epigenetic modifications of the *foxp3* locus allow persistent expression of Foxp3.

The human FOXP3 promoter has recently been mapped to the putative 5′ end of exon-2b, 6.1 kb upstream from the first coding exon [6]. However, other studies have reported promoter activity upstream from exon-1 close to the differentially methylated region analyzed in the current study [26,27] corresponding to the Foxp3 mRNA species AY357712 and AY357713. We could show here by performing luciferase assays that the differentially methylated region itself possesses transcriptional activity. Together these data suggest that the evolutionarily conserved element might belong to an alternative TATA-less promoter, which contributes to the regulation of Foxp3 expression.

The differential methylation status of the *foxp3* locus in Tregs appears to be a new example for epigenetic regulation of cell lineage differentiation. Although almost all cells in an individual contain the same complement of DNA code, higher organisms must impose and maintain different patterns of gene expression in the various types of differentiated cells. Most gene regulation is transitory, depending on the current state of the cell and changes in external stimuli. Persistent regulation, on the other hand, is a primary role of epigenetics: heritable regulatory patterns that do not alter the basic genetic coding of the DNA. DNA methylation is the archetypical form of epigenetic regulation; it serves as the stable memory for cells and performs a crucial role in maintaining the long-term identity of various cell types. Our finding that evolutionarily conserved sequences within the *foxp3* locus are completely and selectively demethylated upon differentiation into persistent Tregs suggests an important role of epigenetic fixation for this phenotype.

Moreover, this seems to be the first report that a transcription factor acting as a master switch for a certain subpopulation is itself subject to epigenetic control. The role of transcription factors such as T-bet or GATA-3 for the polarization of Th1 and Th2 cells, respectively, has been carefully studied, and their interplay with epigenetically regulated regions in the respective cytokine genes is seen as a major factor in the acquisition of cytokine memory [18,19]. However, *foxp3* appears to be the first known example, at least in the immune system, in which the transcriptional regulation of a master transcription factor itself involves epigenetic mechanisms.

A crucial finding of this study is that chromatin remodeling of the *foxp3* locus does not merely correlate with Foxp3 expression. Rather, our current data provide first experimental evidence that the completely demethylated status of the evolutionarily conserved region is only confined to stable Treg populations, such as the naturally occurring, thymus-derived CD25^+^CD4^+^ cells, and might indeed be a prerequisite for the permanent commitment to the suppressor cell lineage. This assumption is based on the analysis of TGF-β–induced Tregs, which, despite Foxp3 expression and suppressive properties, have not acquired a terminally differentiated phenotype and have lost both Foxp3 expression and suppressive capacity upon restimulation in the absence of TGF-β. This indicates that the recently postulated positive autoregulatory loop involving up-regulation of endogenous TGF-β expression and subsequent Foxp3-dependent down-regulation of Smad7, a negative regulator of TGF-β signaling, is not sufficient to induce stable Foxp3 expression in vitro [15]. As TGF-β–induced Tregs display only weakly demethylated CpG motifs within the conserved region of the *foxp3* locus, a more complete CpG demethylation might be the key for a stable Foxp3 expression, similar to what has recently been reported for IL-2 [23].

The findings of this study have important implications with respect to clinical applications. First, determination of the methylation status might allow a better identification and quality control of Tregs considered for cellular therapy concepts of autoimmune diseases, graft-versus-host diseases, or transplant rejections. Temporary expression of FOXP3 can be detected in activated T cells lacking regulatory function, especially in the human system [28,29]. Analysis of the methylation status of the *foxp3* locus promises to be a more reliable marker for the successful conversion of conventional CD4^+^ T cells into a stable population of suppressor cells.

Second, detection of demethylated *foxp3* sequences might allow the development of novel diagnostic tools for the quantification of Tregs in blood or tissues. Both in autoimmune disease and tumor patients, a correlation between Treg number and/or activity and disease status has been observed in a number of recent studies. Decreased activity and/or number of Tregs has been noted to be associated with myasthenia gravis, autoimmune polyglandular syndrome type II, ulcerative colitis, and multiple sclerosis [30–36]. In contrast, an increased number of Tregs was observed in patients with a variety of malignant cancers [37–39], and might be involved in tumor progression [40–42]. Most notably, presence of Tregs, as defined by gene expression of FOXP3, has been shown to constitute a significant predictive parameter for the clinical outcome in ovarian cancer patients [40,41]. However, the analytical value of flow cytometry, immunohistological, and mRNA expression analysis of CD25 and Foxp3 as accurate diagnostic tools is blurred by both ambiguity of the markers and instability of the biological materials. In contrast, our current data show that DNA demethylation at the *foxp3* locus, both in mice and humans, strictly coincides with the generation of stable Tregs. Therefore, measurement of the methylation status of the *foxp3* locus could present a more reliable and objective criterion for the identification and quantification of Tregs. Moreover, DNA methylation is intrinsically a more stable parameter than mRNA expression or protein synthesis, and can be accurately quantified [43]. Therefore, we believe that establishment of a measurement system for the methylation status of the human *foxp3* locus may provide a novel diagnostic tool both in tumor and in autoimmune disease patients.

## Materials and Methods

### Mice.

BALB/c mice were bred at the BfR (Bundesinstitut fuer Risikobewertung, Berlin, Germany) and used at 6–12 wk of age. All animal experiments were performed under specific pathogen-free conditions and in accordance with institutional, state, and federal guidelines.

### Antibodies, staining reagents, and cytokines.

The following antibodies were produced in our laboratory: anti-FcR II/III (2.4G2), anti-CD4 (GK1.5), anti-CD3 (145.2C11), anti-CD28 (37.51), and anti–IL-4 (11B11). The following antibodies and secondary reagents were purchased from BD PharMingen (San Diego, California, United States): anti-CD4 (RM4–5), anti-CD19 (1D3), anti-CD25 (7D4), anti-CD8 (53–6.7), anti-CD25 (PC6.1), anti-CD62L (Mel-14), streptavidin, and appropriate isotype controls. The PE anti-mouse Foxp3 staining set was purchased from eBioscience (San Diego, California, United States). All microbeads were obtained from Miltenyi Biotec (Bergisch Gladbach, Germany) and all cytokines from R & D systems (Minneapolis, Minnesota, United States).

### Flow cytometry.

Cytometric analysis was performed as previously described [44] using a FACS Calibur or a LSRII (BD Biosciences, Palo Alto, California, United States) and the CellQuest software. Dead cells were excluded by PI (propidium iodide) or DAPI (diamidophenylindole) staining (Sigma, St. Louis, Missouri, United States). Intracellular Foxp3 staining was performed with the PE anti-mouse Foxp3 staining set according to the manufacturer's instructions.

### Ex vivo cell sorting.

CD4^+^ T cells were isolated from pooled spleen and lymph node (LN) single-cell suspensions by using anti–CD4-FITC plus anti-FITC multisort microbeads and the AutoMACS magnetic separation system (Miltenyi Biotec). After release of beads according to the manufacturer's instructions, CD25^+^ and CD25^−^ cells were separated using anti–CD25-APC plus anti-APC microbeads. Thymic single-cell suspensions were sorted for CD8^+^ and CD8^−^ cells using anti-CD8 microbeads and the AutoMACS magnetic separation system. MACS-sorted CD8^−^ thymocytes were subsequently stained using anti–CD4-FITC, anti–CD25-APC, and anti–CD19-PE, and sorted into CD25^+^ and CD25^−^ subsets of CD4 SP thymocytes as well as into CD19^−^ DN thymocytes by fluorescence-activated cell sorting (FACS) (FACSAria; BD Bioscience). Magnetic-activated cell sorting (MACS)-sorted CD8^+^ thymocytes were stained using anti–CD4-FITC and anti–CD8-PerCP, and sorted into DP thymocytes by FACS. All subsets were sorted to a purity of greater than 98%.

### Adoptive transfer of sorted CD25^+^CD4^+^ T cells.

Sorted CD25^+^CD4^+^ T cells were labeled with CFSE (Molecular Probes, Eugene, Oregon, United States) as described before [45]. A total of 2 × 10^6^ CFSE-labeled cells were adoptively transferred into syngenic recipients. Fourteen days after transfer, single-cell suspensions of spleen, peripheral, and mesenteric LNs were prepared and stained for CD4, CD25, and Foxp3 as described above.

### TGF-β–induced Tregs.

CD4^+^ T cells were isolated from pooled spleen and LN single-cell suspensions by using anti–CD4-FITC plus anti-FITC multisort microbeads and the AutoMACS magnetic separation system (Miltenyi Biotec). After release of beads according to the manufacturer's instructions, CD25^+^ cells were depleted by using anti–CD25-APC plus anti-APC microbeads. To avoid the expansion of precommitted Foxp3^+^ Tregs, we excluded the majority of residual Foxp3^+^ Tregs from the CD25^−^CD4^+^ T cell fraction by sorting for CD62L^high^ cells using anti-CD62L microbeads. MACS-sorted CD62L^high^CD25^−^CD4^+^ T cells were stimulated for 3 d using plate-bound anti-CD3 (6 μg/ml) and anti-CD28 (4 μg/ml). For Th1 cultures, 5-μg/ml anti–IL-4, 20-ng/ml IFN-γ and 5-ng/ml IL-12 were added to the medium. For TGF-β cultures, 5-ng/ml TGF-β and 10-ng/ml IL-2 was used. After 3 d, cells were removed from the stimulus, transferred to non-coated plates, and cultured for another 3 d. All cell culture was done with RPMI 1640 (GIBCO, San Diego, California, United States) supplemented with 10% FCS (Sigma). On day 6, cultured cells were stained using anti–CD25-APC and sorted for CD25^+^ cells by FACS (FACSAria). Foxp3 expression of sorted CD25^+^ cells was analyzed by intracellular staining. For restimulation experiments, TGF-β–induced Tregs were sorted for CD25^+^ cells by FACS (FACSAria), and sorted CD25^+^ cells were stimulated for 3 d using plate-bound anti-CD3 (6 μg/ml) and anti-CD28 (4 μg/ml) plus IL-2 (10 ng/ml). After 3 d, cells were removed from the stimulus, transferred to non-coated plates, and cultured for another 3 d. On day 6, cultured cells were stained for CD25 and Foxp3 as described above.

### Culture of ex vivo isolated CD25^+^CD4^+^ Tregs.

CD25^+^ cells were enriched from pooled spleen and LN single-cell suspensions by using anti–CD25-FITC, anti-FITC microbeads, and the AutoMACS magnetic separation system (Miltenyi Biotec). MACS-enriched CD25^+^ T cells were subsequently stained using anti–CD4-PerCP and anti–CD62L-APC, and sorted for CD62L^high^CD25^+^CD4^+^ Tregs by FACS (FACSAria). CD62L^high^ Tregs were used to avoid the expansion of Foxp3^−^CD25^+^ effector T cells. FACS-sorted CD62L^high^CD25^+^CD4^+^ Tregs were stimulated for 3 d using plate-bound anti-CD3 (6 μg/ml) and anti-CD28 (4 μg/ml) plus IL-2 (40 ng/ml), followed by transfer to non-coated plates and culture for another 3 d. On day 6, cultured cells were stained for CD25 and Foxp3 as described above.

### In vitro suppression of naïve CD4^+^ T cell proliferation.

The assay was performed as previously described [45]. Proliferation of naïve CD62L^high^CD25^−^CD4^+^ responder cells was evaluated according to CFSE dilution.

### Bisulphite sequencing.

Genomic DNA was isolated from purified T cells using the DNeasy tissue kit (Qiagen, Valencia, California, United States) following the supplier's recommendations. Sodium bisulphite treatment of genomic DNA was performed according to Olek et al. [46] with minor modifications, resulting in the deamination of unmethylated cytosines to uracil, whereas methylated cytosines remain unchanged. In a subsequent PCR amplification, uracils were replicated as thymidines. Thus, detection of a “C” in sequencing reactions reflects methylation of the genomic DNA at that site. Detection of a “T” at the same site reflects instead the absence of a methyl modification of the genomic cytosine.

PCRs were performed on MJ Research thermocyclers (Waltham, Massachusetts, United States) in a final volume of 25 μl containing 1× PCR Buffer, 1-U Taq DNA polymerase (Qiagen), 200 μM dNTPs, 12.5 pmol each of forward and reverse primers, and 7 ng of bisulphite-treated genomic DNA. The amplification conditions were 95 °C for 15 min and 40 cycles of 95 °C for 1 min, 55 °C for 45 sec, and 72 °C for 1 min, and a final extension step of 10 min at 72 °C. PCR products were purified using ExoSAP-IT (USB Corp, Staufen, Germany) and sequenced in both directions applying the PCR primers and the ABI Big Dye Terminator v1.1 cycle sequencing chemistry (Applied Biosystems, Foster City, California, United States), followed by capillary electrophoresis on an ABI 3100 genetic analyzer. Trace files were interpreted using ESME, which normalizes sequence traces, corrects for incomplete bisulphite conversion, and allows for quantification of methylation signals [47]. For each sample, both PCR amplification and sequencing was repeated once. The following primers (5′ to 3′ direction) were used for both PCR amplification of bisulphite converted genomic DNA and sequence reactions: Amp 1 (fw: AGGAAGAGAAGGGGGTAGATA; rev: AAACTAACATTCCAAAACCAAC), Amp 2 (fw: ATTTGAATTGGATATGGTTTGT; rev: AACCTTAAACCCCTCTAACATC), Amp 3 (fw: AGAGGTTGAAGGAGGAGTATTT; rev: ACTATCTATCCAATTCCCCAAC), and Amp 4 (fw: TGGTTGTTTTGGAGTTTAGTGT; rev: CACTTTTCTACCTTCCACAAAT).

### Construction of the luciferase reporter vector.

The differentially methylated, conserved element (CE) of the *foxp3* locus was amplified by PCR using the mouse BAC RPCIB731D08143Q2 (RZPD) as a template and the following primers: 5′-GATCGGTACCTTGTCCCAGGAGAGCGGG-3′, and 5′-GATCCCCGGGCCCATATGGCTGGACCATGG-3′.

The amplified 1,160-bp element was cloned via Asp718 and XmaI into the pGL3 promoter vector (Promega, Madison, Wisconsin, United States) in front of the minimal SV40 promoter to generate pGL3-Foxp3-CE.

### Luciferase assay.

RLM-11–1 cells [48], which were kindly provided by Marc Ehlers (Deutsches Rheuma-Forschungszentrum [DRFZ], Berlin, Germany), or ex vivo isolated CD25^+^CD4^+^ Tregs were transfected using 4 μg of pGL3 promoter vector (control) or pGL3-Foxp3-CE. Synthetic Renilla luciferase reporter vector (pRL-TK, 0.2 μg; Promega) was used as an internal control for transfection efficiency. Four hours after transfection via nucleofection (Amaxa Cologne, Germany), RLM-11–1 cells were stimulated with PMA (10 ng/ml; Sigma) or with PHA (20 ng/ml; Sigma) plus ionomycin (1 μM; Sigma) in case of ex vivo isolated Tregs. After 18–24-h culture in IMEM (GIBCO), cells were harvested and luciferase activity was measured using the dual luciferase assay system (Promega). Data were normalized to the Renilla luciferase activity.

### ChIP.

ChIP analysis was carried out according to the manufacturer's protocol (Upstate/Millipore, Billerica, Massachusetts, United States). Cells (1–5 × 10^6^) were fixed with 1% formaldehyde, and chromatin was fragmented by ultrasound. For all ChIP samples, sheared chromatin was precleared by incubation with ProteinA-agarose/salmon sperm DNA (Upstate/Millipore). Subsequently, chromatin was immunoprecipitated by overnight incubation at 4 °C with 4-μg antibodies (rabbit isotype, #2027, Santa Cruz Biotechnology [Santa Cruz, California, United States]; anti–acetyl-histone H3, #06–599, Upstate/Millipore; anti–acetyl-histone H4, #06–866, Upstate/Millipore; and anti–trimethyl-K4-histone H3, #07–473, Upstate/Millipore) followed by incubation with Protein A-agarose/salmon sperm DNA for 1 h. Precipitates were defixed and DNA was purified by using the NucleoSpin Extract II kit (Macherey-Nagel, Düren, Germany). The amount of immunoprecipitated DNA was quantified by real-time PCR with LightCycler (Roche Applied Science, Basel, Switzerland) using SYBR Green and the following primer pair (5′ to 3′ direction): Foxp3 (331 bp) fw: GACTCAAGGGGGTCTCA; rev: TTGGGCTTCATCGGCAA.

Sample PCR products were set in relation to the input DNA using the following expression: 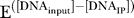 .

### Bioinformatics.

Genomic sequences spanning the *foxp3* locus were analyzed using the alignment software vista (http://pipeline.lbl.gov/servlet/vgb2), allowing the identification of conserved regions. Transcription factor binding sites were identified using the TRANSFAC database [49] and the search tool MATCH [50].

## Supporting Information

Figure S1 CpG Motifs and Transcription Factor Binding Sites within Differentially Methylated, Conserved Element of the *foxp3* Locus Alignment of mouse (upper row) and human (lower row) genomic sequences corresponding to amplicons 1 and 2 of the *foxp3* locus. Individual CpG motifs are shown in red, and putative transcription factor binding site core elements, which were identified using the TRANSFAC database and the search tool MATCH, are illustrated as boxes.
(25 KB PDF)

Figure S2 Suppressive Capacity of TGF-β–Induced Tregs CD25^−^CD4^+^ T cells were cultured essentially as described in Figure 6. Cultured cells were incubated with CFSE-labeled naïve CD25^−^CD62L^high^ CD4^+^ responder T cells at a ratio of 1:1 in the presence of CD90-depleted APCs and anti-CD3 for 3 d. Proliferation of naïve responder cells was evaluated according to CFSE dilution. Histograms show CFSE staining of responder T cells cultured alone (positive) or cocultures with ex vivo isolated CD25^+^CD4^+^ Tregs (ex vivo), with T cells cultured for 6 d under neutral conditions (neutral), with T cells cultured for 6 d in the presence of TGF-β (TGF-β), with T cells cultured for 6 d under neutral conditions and restimulated under neutral conditions (neutral/neutral), and with T cells cultured for 6 d in the presence of TGF-β followed by restimulation under neutral conditions (TGF-β/neutral). Representative data from one out of three independent experiments with similar results are depicted.
(72 KB PDF)

Table S1 Methylation Status of Individual CpG Motifs within the *foxp3* Locus Summary of all DNA methylation analyses performed for indicated subsets. Shown is the degree of methylation (%) for individual CpG motifs. CpG motifs from amplicon 2 overlapping with motifs in amplicon 1 were excluded. n.a., not analyzed.
(72 KB PDF)

### Accession Numbers

The GenBank (http://www.ncbi.nlm.nih.gov/Genbank) accession number for the forkhead transcription factor Foxp3 is AF277994.

## Abbreviations

bp: base pair
ChIP: chromatin immunoprecipitation
FACS: fluorescence-activated cell sorting
IL: interleukin
kb: kilobase
LN: lymph node
SP: single positive
Treg: regulatory T cell
