# Supplementary material for: Epigenetic Control of the foxp3 Locus in Regulatory T Cells
Source: PLoS Biol. 2007 Jan 30;5(2):e38. doi: 10.1371/journal.pbio.0050038 (PMC1783672; doi:10.1371/journal.pbio.0050038)
Supplement: Supplementary file 1 [file pbio.0050038.sg001.pdf]

```
   1 AGGCTGACATTCCAGAGCC-----AGCAAGAGGCCTATGGAGTTTTAAG      45
         ||   .|||||| |||      .||||||||||.||||.||....|||
   1 ----tg--tttccag-gcctggtaggcaagaggccctatgaagcagcaag      43

                          NF/kappaB  GATA-4
  46 CTTCCTGGCTTT-AGGTGGTTCCCATTTCTTTGGGC-TCTGGGACA----      89
     ||.||||.|||| ||.|||||||.|....|||||.| .|.||||||
  44 ctgcctgactttcagatggttccaaggagtttggacaccagggacactgg      93

    Foxp3
  90 TCAATACACACAGTAAGAAGGTGGATCCATGCACCCTACAGAGTCTGTGT     139
     .|  ||||||.|.|.|||...|||..||.|..||||  ||.||||||||.
  94 cc--tacacatactgagactttgggaccgtagaccc--cacagtctgtgg     139

                 C/EBPgamma    TCF-4  C/EBPgamma
 140 TCTTGAGATTCTAAAATCCGTTGGCTTTGAGAAATGATATCGTACAGTTC     189
     |.|||||||||||..||||.||...|.|.|||||||.|.|..||...|||
 140 ttttgagattctaggatcctttaaatctaagaaatgctgttctatgattc     189

                                 TTF1/TCF-4
 190 TGAGTTTCTGTTACTACAGCATTTGAAGACTCAAGGGGGTCTCAATATCC     239
     ||||.|.|||.|..||.|..||||||||||.|.| ||||||.||.||||.
 190 tgaggtcctggtgttatactatttgaagacccca-ggggtcccagtatct     238

 240 ATGAGGCCTGCCTAATACTCACCAAGCATCCAACCTTGGGCCCCTCTGGC     289
     .||..|||||||||...||||.|..|||.||.|||||.||.||.|||....
 239 gtggagcctgcctggcactctcagagcttcaaacctgggtcctctccaca     288

 290 ATCCAAGAA-AGACAGAATCGATAGAACTTGGGTTTTG-CATGGTAGCCA     337
     |.|||||||| .|.||| .||...|||.||.|||..||| |||.||.||||
 289 acccaagaagggccag-gtcttcagagctaggggcttgtcatagtggcca     337

   GATA-4 ATF/CREB    c-Ets-1
 338 GATGGACGTCACCTACCACATCCGCTAGCACCCACATCACCCTACCTGGG     387
     |||||||.||||||||||||||||.|.|||||||||..||||||.|||||||
 338 gatggacatcacctaccacatccaccagcacccatgtcaccccacctggg     387

     NF-kappaB                               STAT-1
 388 CCTATCCGGCTACAGGATAGACTAGCCACTTCTCGGAACGAAACCTGTGG     437
     ||.|.||.|||.|||||.||...|||||.||||||||||||||||||||||
 388 ccaagcctgctgcaggacagggcagccagttctcggaacgaaacctgtgg     437

 438 GGTAGATTATCTGCCCCCTTCTCTTCCTCCTTGTTGCCGATGAAGCCCAA     487
     |||.|..||||||||||.|||||||||||||.||.||.||||||||||||..
 438 ggtggggtatctgccctcttctcttcctccgtggtgtcgatgaagcccgg     487

       c-Ets-1      CREB/ATF
 488 TGCATCCGGCCGCCATGACGTCAATGGCAGAAAAATCTGGCCAAGTTC--     535
     .|||||||||||||||||||||||||||.||||||||||||.|||| ||
 488 cgcatccggccgccatgacgtcaatggcggaaaaatctgggcaag-tcgg     536

            Foxp3                                NFAT/Evi-1
 536 AGGTTGTGACAACAGGGCCCAGATGTAGACCCCGATAGGAAAACATATTC     585
     .||.|||||||||||||||||||||.||||||||||||.||||||||||.||
 537 gggctgtgacaacagggcccagatgcagaccccgatatgaaaacataatc     586

 586 TATGTCCCAGAAACAACCTCCATACAGCTTCTAAGAAA--CAGTCAAACA     633
     |.||||||||||||||.||.||||.|||||||||.|||||  ||||||.|.|
 587 tgtgtcccagaaacatccccattcagcttctgagaaacccagtcagaaa     636

                               Elk-1  SMAD-4
 634 GGAACGCCCCAACAGACAGTGCAGGAAGCTGGCTGGCCAGCCCAGCCCTC     683
     ||.|||.|||||||||||||||||||||.||||||.||||||||.||||||
 637 gggacgtcccaacagacagtgcaggaagccggctgcccagcccggccctc     686

                              SMAD-4
 684 CAGGTCC-CTAGTACCACTAGACAGACCATATCCAATTCAGG--          724
     .|||||| |   ||||.|.||||||||.|||.|||||..||...
 687 taggtcctc---taccccagacagatcatctccatgtccctgt           727
```

Supplementary Figure 1
