# Supplementary material for: Epigenetic Control of the foxp3 Locus in Regulatory T Cells
Source: PLoS Biol. 2007 Jan 30;5(2):e38. doi: 10.1371/journal.pbio.0050038 (PMC1783672; doi:10.1371/journal.pbio.0050038)
Supplement: Supplementary file 2 [file pbio.0050038.sg002.pdf]

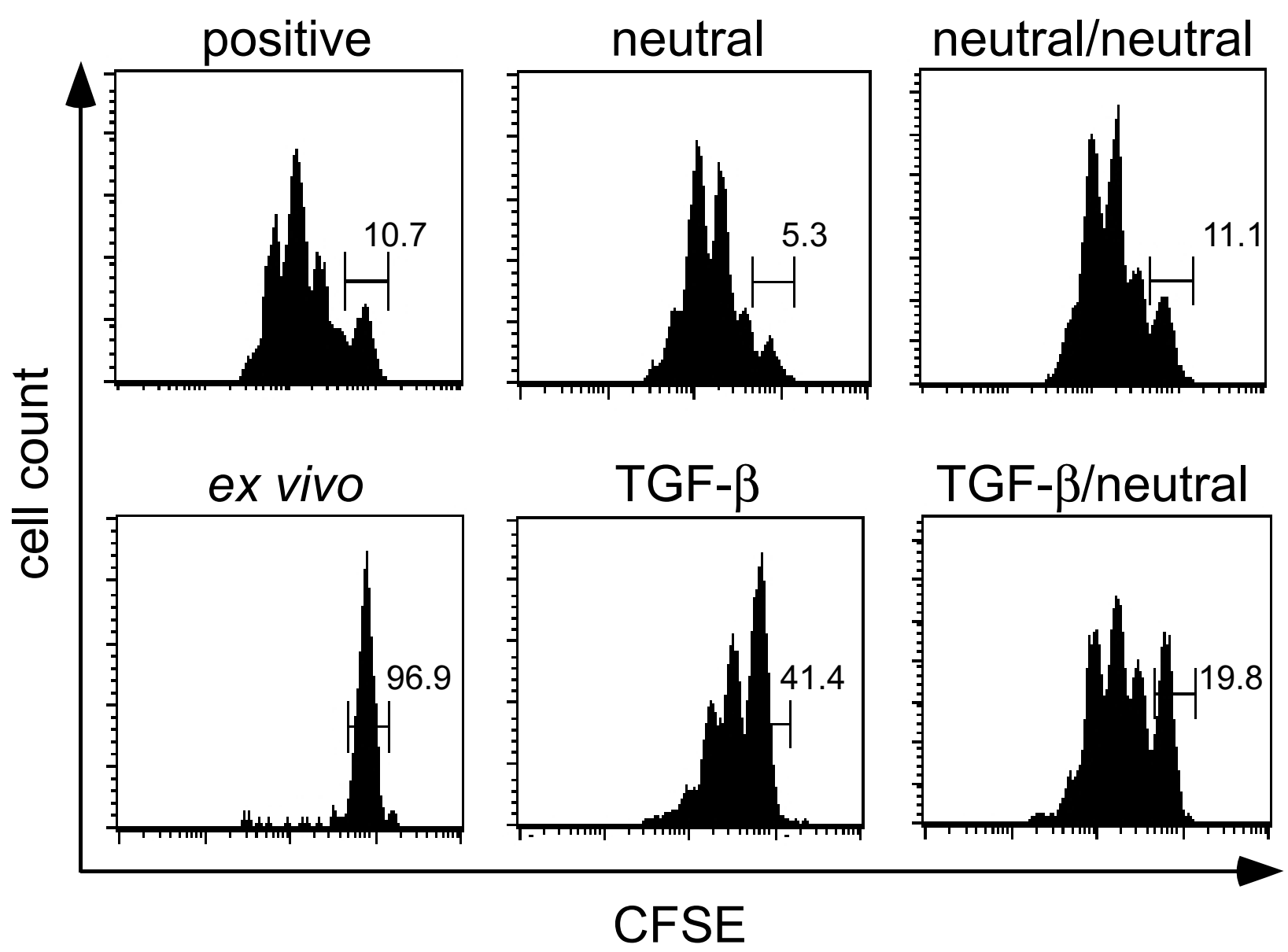

Supplementary Figure 2
