# Supplementary material for: Epigenetic Control of the foxp3 Locus in Regulatory T Cells
Source: PLoS Biol. 2007 Jan 30;5(2):e38. doi: 10.1371/journal.pbio.0050038 (PMC1783672; doi:10.1371/journal.pbio.0050038)
Supplement: Supplementary file 3 [file pbio.0050038.st001.pdf]

Supplementary Table

amplicon (CpG position)	ex vivo periphery				ex vivo thymus						*in vitro*		
					CD4 SP				DP	DN			
	CD25$^-$		CD25$^+$		CD25$^-$		CD25$^+$				Th1	TGF-β	
	exp. 1	exp. 2	exp. 1	exp. 2	exp. 1	exp. 2	exp. 1	exp. 2				exp. 1	exp. 2
1 (158)	100	100	4,5	0	100	100	29	44	100	100	100	100	100
1 (180)	100	100	3,5	12	100	100	40.5	47	100	100	100	92	93
1 (308)	100	100	0	0	100	100	20	33	100	100	100	63	75
1 (344)	100	100	0	0	100	100	38.5	37.5	100	100	100	41	41
1 (360)	100	100	5	0	100	100	34.5	37.5	98	100	100	60	70
1 (394)	100	100	0	6	100	100	12	24.5	100	100	100	28	38
1 (421)	96	100	5	1.5	100	100	23.5	0	100	100	100	42	n.a.
1 (426)	100	96.5	4	2	100	100	29	12	93	100	100	48	n.a.
2 (205)	98.5	100	0	0	100	98	87	36.5	95	100	100	74	67,5
2 (224)	98	100	0	0	100	96	85	95	100	100	100	72	75
2 (228)	100	100	13,5	15	100	100	100	100	100	100	100	100	90
2 (236)	100	100	0	0	100	100	83	73	100	100	100	75	66
2 (298)	98.5	100	0	0	100	100	69.5	50	93.5	100	100	64	43
2 (368)	100	100	0	3.5	100	100	64	47	91.5	100	100	39	50
3 (93)	100	98	57	79.5	100	100	85	100	100	100	92.5	100	n.a.
3 (170)	100	100	50	100	100	100	100	100	100	100	100	100	100
3 (173)	100	100	100	100	100	100	92	100	100	100	100	100	100
3 (176)	100	100	100	100	100	100	100	100	100	100	100	100	100
3 (281)	100	100	87	91.5	100	100	100	100	100	100	100	100	96
4 (37)	n.a.	n.a.	n.a.	n.a.	100	n.a.	n.a.	100	n.a.	n.a.	100	n.a.	n.a.
4 (69)	n.a.	100	n.a.	80	100	100	90	95	95	100	100	100	100
4 (311)	100	100	100	100	100	100	100	100	100	100	100	100	100
4 (315)	100	100	100	100	100	100	100	100	100	100	100	100	100
4 (319)	100	100	100	100	100	100	100	100	100	100	100	100	100
4 (338)	100	100	75	82.5	100	100	100	94.5	100	100	100	90	100
4 (371)	100	100	100	100	100	100	100	97	94	100	100	100	100
